# Real-World Observational Study of Glimepiride and Metformin Fixed-Dose Combination Along With Insulin in the Management of Type 2 Diabetes Mellitus: Indian Experience

**DOI:** 10.7759/cureus.13020

**Published:** 2021-01-30

**Authors:** KM Prasanna Kumar, Krishna Seshadri, SR Aravind, Prasun Deb, KD Modi, Raju A Gopal, Vijaya Kumar G, CR Anand Moses, Mahesh Abhyankar, Santosh Revenkar

**Affiliations:** 1 Department of Endocrinology, Center for Diabetes and Endocrine Care, Bengaluru, IND; 2 Department of Endocrinology, Apollo Speciality Hospital, Chennai, IND; 3 Department of Diabetology, Diacon Hospital, Bengaluru, IND; 4 Department of Endocrinology, Krishna Institute of Medical Sciences (KIMS) Hospital, Hyderabad, IND; 5 Department of Endocrinology, CARE Hospital, Hyderabad, IND; 6 Department of Endocrinology, Endodiab Clinic, Kozhikode, IND; 7 Department of Diabetology, Diabetes Medicare Centre, Chennai, IND; 8 Department of Diabetology, Moses Diabetes and Medical Centre, Chennai, IND; 9 Scientific Services, USV Private Limited, Mumbai, IND

**Keywords:** diabetes, dosage up titration, hba1c, hypoglycemic, tolerability

## Abstract

Background

Type 2 diabetes mellitus (T2DM) is associated with a significant burden on both patients and the healthcare system. This study aimed to evaluate the demographics of patients with T2DM receiving different strengths of glimepiride and metformin combination along with insulin. This study also examined the concomitant conditions and therapies, duration of therapies, dosage titration, glycated hemoglobin (HbA1c) levels, hypoglycemic events, and weight changes during the course of therapy.

Methods

This retrospective, multicenter (347), observational study included adult patients with T2DM who received glimepiride and metformin combination along with insulin. Data related to demographic characteristics, duration of disease, co-morbidities, concomitant medications, and dosage pattern was collected from medical records authenticated by physicians during routine care.

Results

A total of 7058 patients were included in the study. The median age of included patients was 55 years and around 29% were aged >60 years and 60% were men. The majority of patients (83.3%) had insulin treatment initiation after glimepiride and metformin combination while other patients (16.7%) received glimepiride and metformin combination after insulin initiation. The mean HbA1c levels significantly decreased with a mean change of 1.33%. In one-third of the patients, down-titration of the insulin dose was done, indicating the insulin-sparing effect with the addition of the glimepiride and metformin combination. The most common comorbid condition was hypertension (64.7%). Of 3705 patients, 33.2% patients had weight loss and 66.8% had weight gain. A total of 432 patients reported hypoglycemic events. Physician global evaluation of efficacy and tolerability showed a good to excellent on the scale (97.3% and 96.6%).

Conclusion

This study presented good HbA1c lowering with glimepiride and metformin combination with insulin, ensuring a positive clinical outcome. Good to excellent efficacy and tolerability were observed in patients with T2DM across the age groups, in early as well as long-standing disease.

## Introduction

Type 2 diabetes mellitus (T2DM) is predominant in the Asian population, majorly affecting young to middle-aged adults, despite low body mass index (BMI) and obesity. Predictions estimated that the number of patients from Asia with diabetes would upsurge dramatically by 2025 [1]. Additionally, it is also estimated that globally 700 million adults will have diabetes by 2045 [1].

Achieving ideal glycemic control is the key goal to delay complications and advancement to T2DM. Sulfonylurea and insulin are among the commonly used drugs when the glycemic goal is not achieved by using metformin monotherapy, especially in Asian countries [2].

The fixed drug combination of glimepiride and metformin is highly effective in controlling blood glucose and improving glycated hemoglobin (HbA1c) levels than if used as monotherapy [3]. This combination enhances patient compliance and reduces each component drug’s dosage. In addition, the therapy cost and adverse effects due to high dosage monotherapy are lowered [4].

The American Diabetes Association endorses the addition of insulin to metformin therapy and recommends consideration of the early introduction of insulin [5-6]. Nevertheless, most of the physicians are hesitant to initiate and intensify insulin therapy due to the side effects [7]. A combination of insulin with sulfonylurea works well, resulting in improved glucose levels, less weight gain, and tapered insulin dose. Similarly, it gave efficient results with metformin, showing a 10% lowered HbA1c with 29% less insulin use and less hypoglycemia [8-11]. Instead of adding a third oral agent to the glimepiride and metformin combination, the addition of insulin resulted in lowering HbA1c levels, decreasing the need for exogenous insulin and risk of hypoglycemia and weight gain [12-13]. Moreover, studies demonstrate that early glucose control can delay macrovascular and microvascular complications and improve cardiovascular morbidity [14-15]. In Indian settings, the multiple strengths of glimepiride and metformin fixed-dose combinations are beneficial in T2DM, irrespective of age, duration of diabetes, BMI, diabetic complications, and use of concomitant medications such as insulin and statin [16].

Clinical studies have unraveled that combination therapies of oral anti-diabetic drugs and insulin offer complementary mechanisms, leading to improved therapeutic efficacy and minimal adverse reactions [13,17-19]. However, there are no adequate nationwide real-world data analysis in the Indian population on the use of the glimepiride and metformin combination along with insulin. The present real-world study is aimed to evaluate the demographics, treatment pattern, including duration, efficacy, various dosages of the glimepiride and metformin combination along with insulin.

## Materials and methods

Study design

This retrospective, non-randomized, non-comparative, multicenter, observational real-world study of Glycomet glimepiride-insulin (REAL GGP-INS) was conducted at 347 sites in Indian healthcare centers having medical records of adult patients with T2DM who had received treatment with glimepiride and metformin along with insulin.

Data related to demographic characteristics, duration of disease, co-morbidities, concomitant medications, and dosage patterns were collected from medical records authenticated by physicians during routine care.

Study population

Patients of either sex and age above 18 years, who have received any strength of glimepiride and metformin combination along with insulin for the treatment of T2DM and the approval of the treating physician to provide information regarding the participant’s treatment, were enrolled in this study. Patients having incomplete data or any condition that, according to the discretion of the investigator, indicates that the patient is not suitable for inclusion in the study were excluded from the study.

Outcome

The outcome of this observational data analysis was the demographics of patients receiving different strengths of the glimepiride and metformin combination along with different types of insulin, concomitant conditions and therapies, duration of the glimepiride and metformin combination along with insulin therapy, up-titration and down-titration done during the course of therapy, HbA1c levels, hypoglycemic events (mild/moderate/severe/required intervention), and weight change during the course of therapy.

Statistical analysis

Statistical testing was done using appropriate statistical tests. Demographic characteristics were summarized with descriptive statistics, including median and interquartile range (IQR) for continuous variables, and frequency and percentages for categorical variables. The data from all the participating doctors were pooled for analysis and evaluable patient data were analyzed.

## Results

A total of 7058 patients with T2DM were included in this retrospective observational analysis. The median age of patients was 55 years and 29.4% of the study population were in the age group of >60 years. The proportion of male patients (60.1%) was higher than female patients (39.9%).

The median duration of diabetes was significantly increased with increasing age and there was a significant difference between the age group ≥18-≤40 years vs. >40-≤60 years (3 vs. 6 years, p<0.001), >40-≤60 years vs. >60 years (6 vs. 10 years, p<0.001), and group ≥18-≤40 years vs. >60 years (3 vs. 10 years, p<0.001).

Obesity was significantly higher in patients of age group >40-≤60 years (p=0.026), a sedentary lifestyle was significantly higher in patients of age group ≥18-≤40 and >40-≤60 years (p=0.001) while excess alcohol intake higher in patients of age group >60 years (p=0.002) and a family history of diabetes (p<0.001) was more common in patients of age group ≥18-≤40 years as well as >40-≤60 years (Table 1).

**Table 1:** Summary of baseline characteristics according to age Data shown as median (IQR), unless otherwise specified. †n=587; ‡n=4242; §n=2229, unless otherwise specified. BMI, body mass index; IQR, interquartile range; DM, diabetes mellitus. *group A vs B; **group B vs C; ***group A vs C

Characteristics	Group A ≥18-≤40 years (n=587)^†^	Group B >40-≤60 years (n=4242)^‡^	Group C >60 years (n=2229)^§^	Total (N=7058)	P-value
Age (years)	38.0 (35.0-39.0)	52.0 (48.0-56.0)	66.0 (63.0-71.0)	55.0 (48.0-62.0)	<0.001^*,**,***^
Sex, n (%), Men	315 (53.7)	2458 (57.9)	1470 (65.9)	4243 (60.1)	<0.001
BMI (kg/m^2^)	[n=543] 25.8 (23.5-28.9)	[n=3924] 26.7 (24.2-29.6)	[n=2069] 26.8 (24.2-29.8)	[n=6536] 26.7 (24.2-29.7)	<0.001^*,***^, 0.254^**^
DM duration (years)	[n=498] 3.0 (2.0-5.0)	[n=3693] 6.0 (3.0-9.0)	[n=1830] 10.0 (6.0-15.0)	[n=6021] 6.0 (4.0-10.0)	<0.001^*,**,***^
Family history of diabetes	253 (43.1)	1790 (42.2)	743 (33.3)	2786 (39.5)	<0.001
Sedentary lifestyle	237 (40.4)	1719 (40.5)	797 (35.8)	2753 (39.0)	0.001
Obesity	191 (32.5)	1617 (38.1)	816 (36.6)	2624 (37.2)	0.026
Smoking	162 (27.6)	1209 (28.5)	685 (30.7)	2056 (29.1)	0.119
Emotional stress	103 (17.5)	873 (20.6)	430 (19.3)	1406 (19.9)	0.151
Excess alcohol intake	86 (14.7)	542 (12.8)	354 (15.9)	982 (13.9)	0.002
Excess salt intake	79 (13.5)	547 (12.9)	322 (14.4)	948 (13.4)	0.220
Tobacco	39 (6.6)	288 (6.8)	184 (8.3)	511 (7.2)	0.082

The majority of patients (83.3%) had insulin treatment initiation after glimepiride and metformin combination while the other patients (16.7%) received the glimepiride and metformin combination on top of insulin. The HbA1c level at the time of initiation of insulin was between 7.5-9% in 59.4% and >9% in 38.9% of the patients taking the glimepiride and metformin combination while the HbA1c level was >9% and 7.5-9% in 50.2% and 46.8%, respectively, in those who received the glimepiride and metformin combination on top of insulin (Table 2).

**Table 2:** HbA1c level at initiation Data shown as n (%). †n=5618; ‡n=1124; unless otherwise specified HbA1c: glycated hemoglobin

	Insulin treatment initiation after Glimepiride and metformin combination (n=5618)^†^	Glimepiride and metformin combination on top of Insulin (n=1124)^‡^
HbA1c level at initiation (%)	[n=5381]	[n=1078)
≤7.5	90 (1.7)	32 (2.9)
>7.5-≤9	3199 (59.4)	505 (46.8)
>9	2092 (38.9)	541 (50.2)

**Figure 1:**
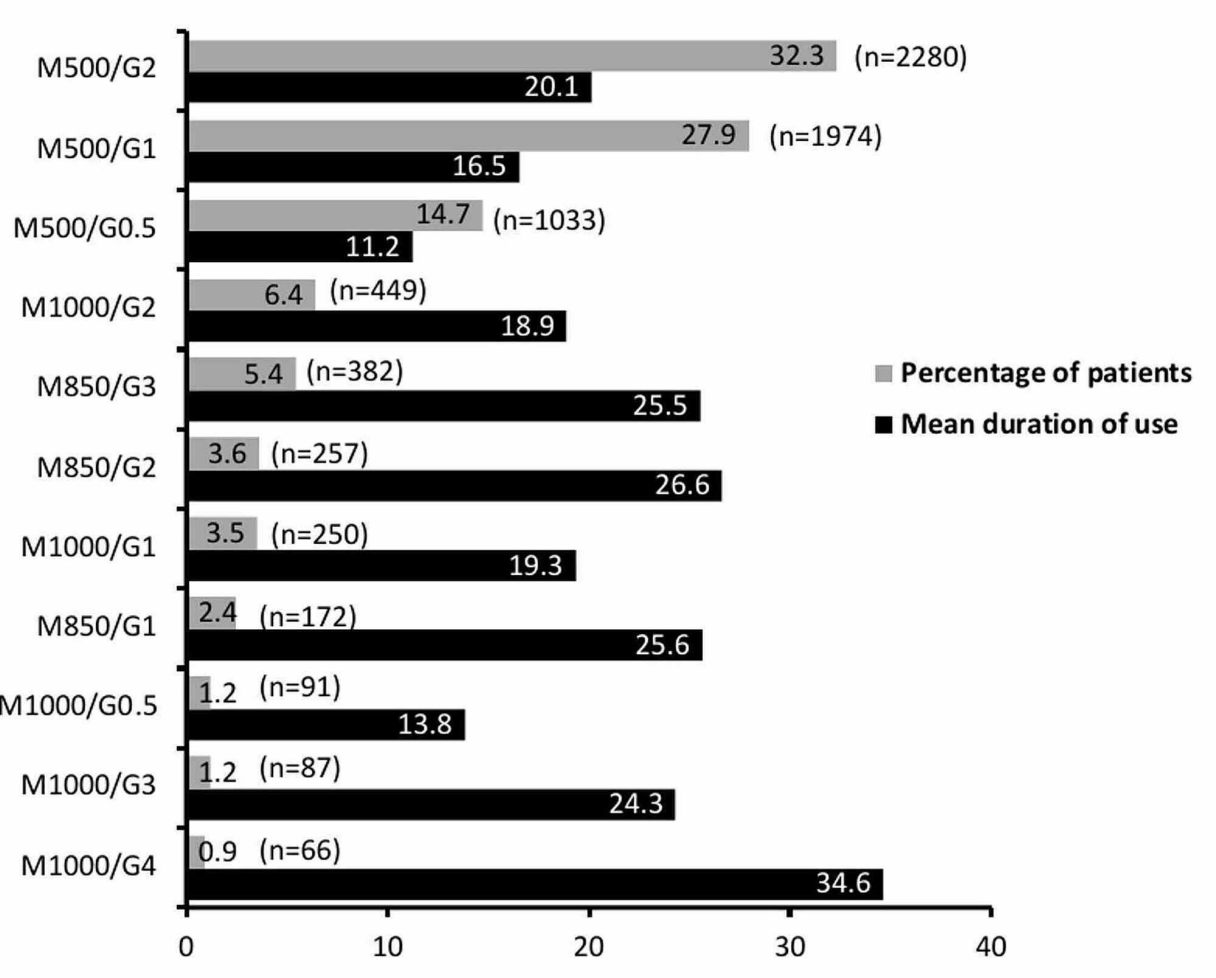
Treatment pattern and duration of use G, glimepiride; M, metformin; compounds presented in mg; duration of use presented in months.

The treatment pattern and frequency are depicted in Figure 1. Along with insulin, the commonest strengths used were glimepiride 2 mg and metformin 500 mg (32.3%) and glimepiride 1 mg and metformin 500 mg (27.9%). The twice-daily (BD) frequency was more than the once-daily (OD). Down-titration of the dose of insulin was required to be done in 34.1% of patients while up-titration was done in 65.9% of patients (Table 3).

**Table 3:** Treatment-related observations Data shown as n (%), unless otherwise specified. †N=7058, unless otherwise specified. AGI, alpha-glucosidase inhibitor; DPP4i, dipeptidyl peptidase-4 inhibitors; GLP1, glucagon-like peptide-1; SGLT2i, sodium-glucose co-transporter-2 inhibitor; TZD, thiazolidinedione. Others, who were on concomitant non-diabetic medication including antiallergic, anticonvulsant, antidepressant, antiepileptic, antifungal, drugs for erectile dysfunction. The intensity of hypoglycemic events was reported based on the treating physician's clinical judgment.

Parameters	Number of patients (N=7058)^†^
Concomitant diabetic medication	[n=3821]
DPP4i	1999 (28.3)
SGLT2i	916 (12.9)
AGI	611 (8.6)
TZD	253 (3.6)
GLP1 agonist	42 (0.6)
Concomitant non-diabetic medication	[n=5999]
Antihypertensive	3061 (51.0)
Statins	890 (14.8)
Drugs in neuropathic pain	199 (3.3)
Others	688 (11.5)
Dose titration of glimepiride and metformin combination	[n=1401]
Dosage up	1268 (90.5)
Dosage down	133 (9.5)
Dose titration of insulin	[n=1782]
Dosage up	1175 (65.9)
Dosage down	607 (34.1)
Patients with weight changes during the therapy	[n=3705]
Increased weight (kg) [n=2474]	
0-2	1719 (69.5)
2-4	669 (27.0)
>4	3.5)
Decreased weight (kg) [n=1231]	
0-2	653 (53.0)
2-4	456 (37.1)
>4	122 (9.9)
Hypoglycemic events	432 (6.1)
Intensity reported [n=429]	
Mild	339 (79.0)
Moderate	85 (19.8)
Severe	3 (0.7)
Required intervention	2 (0.5)

**Figure 2:**
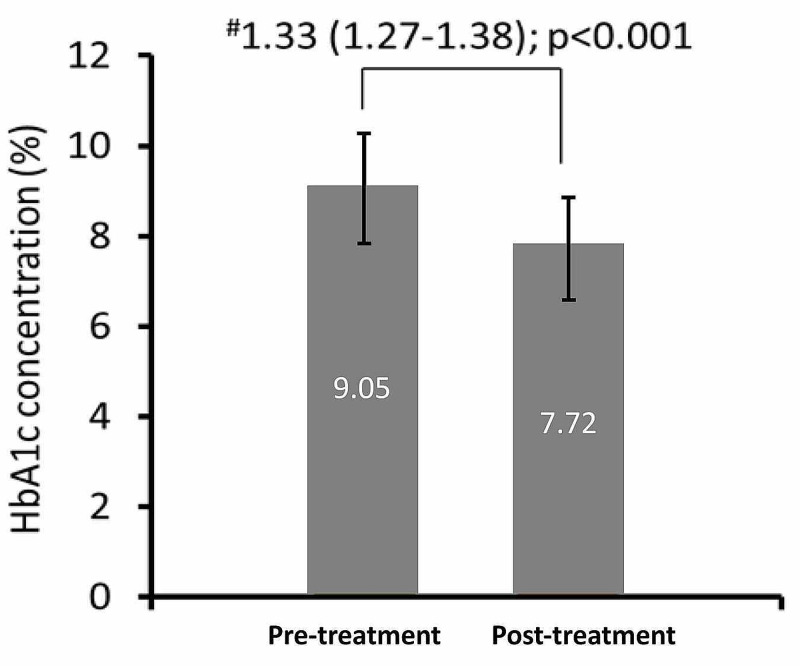
Mean change in HbA1c levels from pre-treatment to post-treatment #Mean change from pretreatment to posttreatment (95% CI); p-value HbA1c: glycated hemoglobin

The mean HbA1c levels significantly decreased post-treatment with the glimepiride and metformin with insulin combination with a mean change of 1.33% (95% CI, 1.27-1.38; p<0.001) (Figure 2).

**Figure 3:**
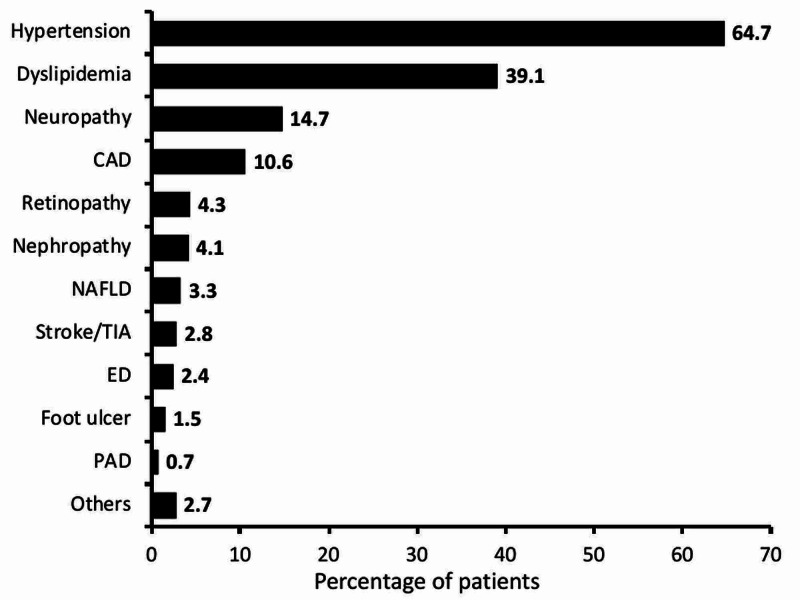
Comorbidities associated with T2DM T2DM, type 2 diabetes mellitus; CAD, coronary artery disease; ED, erectile dysfunction; NAFLD, non-alcoholic fatty liver disease; PAD, peripheral arterial disease, TIA, transient ischemic attacks

In concomitant anti-diabetic medications, the proportion of patients receiving dipeptidyl peptidase-4 inhibitors (DPP4i) (28.3%) was the highest followed by sodium-glucose co-transporter-2 inhibitors (SGLT2i) (12.9%), alpha-glucosidase inhibitor (AGI) (8.6%), thiazolidinedione (TZD) (3.6%), and glucagon-like peptide-1 (GLP1) agonist (0.6%) (Table 3). The common comorbid conditions included hypertension (64.7%), dyslipidemia (39.1%), followed by neuropathy (14.7%), and coronary artery disease (10.6%) across the study population (Figure 3).

**Figure 4:**
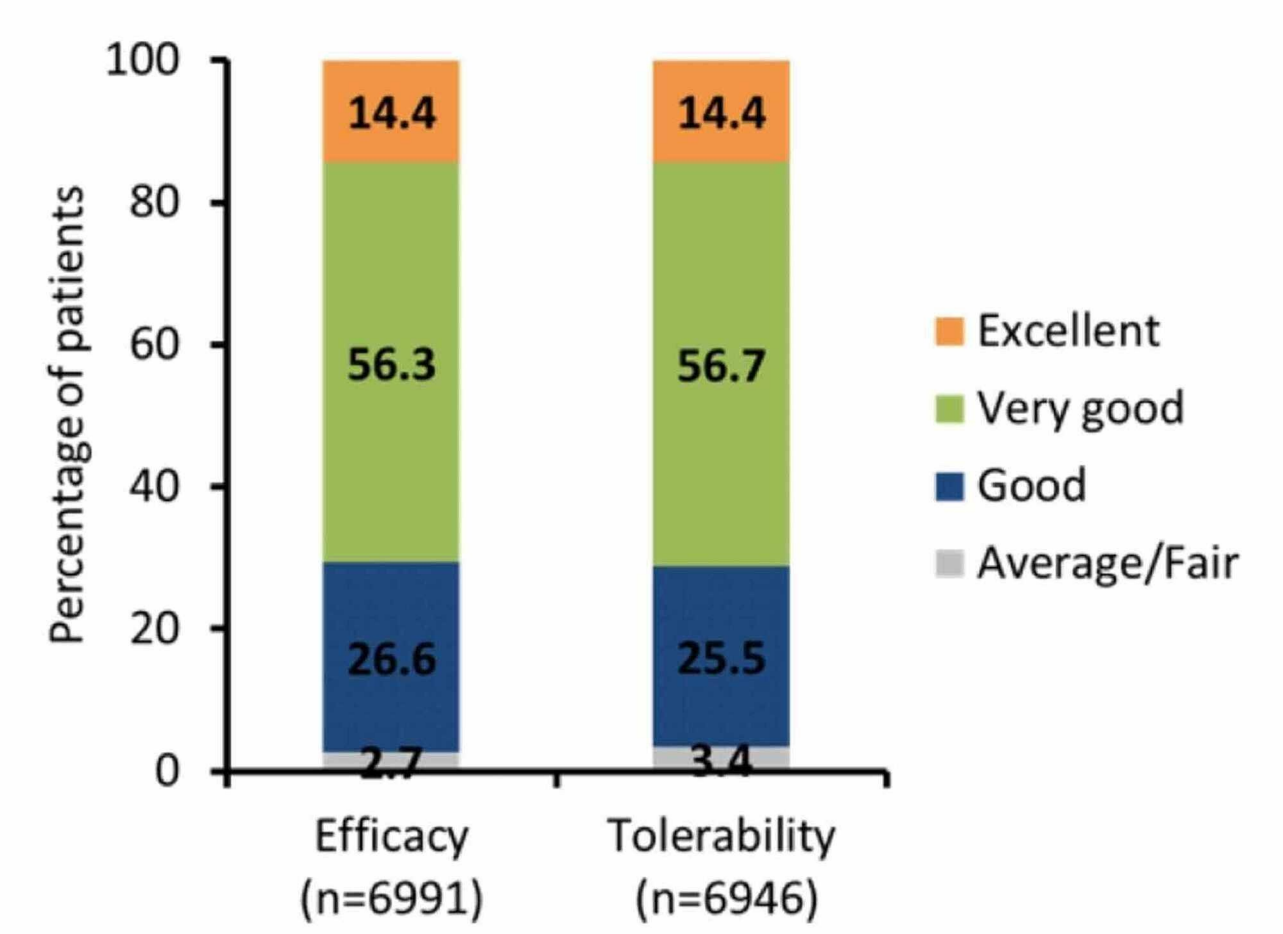
Physician global evaluation of efficacy and tolerability of the glimepiride and metformin combination

A total of 3705 patients experienced weight changes during the treatment, of which 33.2% patients showed weight loss and 66.8% weight gain. The majority of the patients had weight elevation (69.5%) or reduction (53.0%) of up to 2 kgs. A total of 432 patients reported hypoglycemic events. Among them, 79.0% of patients had mild and 19.8% of patients had moderate hypoglycemic events (Table 3). The physician's global evaluation of efficacy and tolerability showed the majority of patients on a good to excellent scale (97.3% and 96.6%) (Figure 4).

## Discussion

T2DM often presents late, and about 70% of the diagnosed cases from India remain uncontrolled [17,20]. Combination therapies involving oral anti-diabetics and insulin have enabled accomplishing a better glycemic index and improve clinical outcomes, resulting in lowering the health complications caused by diabetes. The present study evaluated the use of the glimepiride and metformin combination with insulin to control T2DM in three different age groups: ≥18-≤40 years, >40-≤60 years, and >60 years. Moreover, associated conditions and therapies, duration of the therapy, up- and down-titration, glycemic control, hypoglycemic events, and impact on weight were evaluated. This real-life study conducted in outpatient practices will be an important step towards validating the impact of this treatment pattern.

The key findings of this study showed that obesity, sedentary lifestyle, family history, and emotional stress were remarkably higher in patients of age group >40-≤60 years. However, family history was more common in the age group ≥18-≤40 years. Family history for diabetes, sedentary lifestyle, obesity, smoking, and emotional stress increases the chances of acquiring T2DM [21-22]. Family history is an important predictor. The findings of the current study corroborate with the reported study demonstrating a strong association between age, BMI, and smoking with a prevalence of diabetes [23]. Similarly, another study from Africa showed an incidence of hypertension (71%; 95% CI 69-73), hyperlipidemia (34%), and obesity (27%), along with cataract (32%), diabetic retinopathy (15%), impaired renal function (13%), and erectile dysfunction (in men; 35%) [24-25]. In the current study, hypertension (64.7%) and dyslipidemia (39.1%) were the most common comorbidities observed and managed with antihypertensives and statins, respectively. The observations are in accordance with the reported literature showing the predominance of these comorbidities in India as well as globally.

The mean change in HbA1c levels from pre-treatment to post-treatment with insulin and the glimepiride and metformin combination was significant, indicating good glycemic control.

Reports from the meta-analysis suggest a reduction of HbA1c with insulin‑sulphonylurea combination therapy compared with insulin monotherapy (95% CI: −1.6 to −0.5; p < 0.01) [26], as well as with placebo (95% CI: 0.24, 0.69, I 2 = 43.6%) [27]. A Korean clinical study by Park et al., of a 24 weeks' period, reported a remarkable reduction of HbA1c with the addition of glimepiride to insulin glargine and metformin as compared to insulin glargine plus metformin (0.49% [95% CI: 0.16%-0.82%], P = 0.005) [13]. Another randomized placebo-controlled study conducted in patients with T2DM for more than 10 years duration, who were on metformin and insulin therapy, had glimepiride added, and an effective lowering in HbA1c level with minor hypoglycemic events even after the long duration of T2DM was observed [12]. These studies support the findings from the current study offering an effective therapeutic option for patients not responding to the combination or other drug combinations. Newly diagnosed patients successfully attain glycemic control with monotherapy or a multiple oral drug combination. Nevertheless, over the long term, a strengthened and multidrug regimen becomes a requisite to attain a satisfactory glycemic goal that could be improved by the inclusion of insulin [18].

Dosage up- and down-titration had to be done with the glimepiride and metformin combination as well as insulin to control hyperglycemia, hypoglycemia, and glycemic variability. In one-third of the patients, down-titration of the insulin dose was done, indicating the insulin-sparing effect, with the addition of the glimepiride and metformin combination.

Weight changes are expected in T2DM patients and also due to insulin treatment. Surprisingly, 48% of patients showed no change in weight. Among the remaining patients, 33.2% patients showed weight loss while 66.8% showed weight gain. Glimepiride being modern sulphonylurea has a low propensity to cause weight gain. Metformin use is associated with a decrease in weight if given as a monotherapy or even in combination with insulin or sulfonylurea, which seems to counterbalance the weight gain effect of other anti-diabetic agents [28-29]. In the current study, the sedentary lifestyle observed in all age groups may be one of the contributors associated with weight gain.

Indian clinicians are very accustomed to using the glimepiride and metformin combination along with insulin. Physician global evaluation of efficacy and tolerability showed the majority of patients on a good to excellent scale (97.3% and 96.6%).

This was a real-world study depicting the clinical experience with the administration of various strengths of the glimepiride and metformin combination with insulin given in all age groups, including the elderly. Moreover, this combination with insulin was administered to patients with co-morbidities, including hypertension, dyslipidemia, and coronary artery diseases. A high perception of efficacy and tolerability was observed with this combination.

Limitations

Although this study included a large patient population, it is limited due to its retrospective nature. Besides this, T2DM cases included in this study were enrolled from Indian healthcare centers, which may limit the extrapolation of these findings to the general population. The measurement of fasting and postprandial blood glucose levels was not recorded. Additionally, missing data of patients who failed to report has limited the analysis strength of the study parameters.

## Conclusions

The add-on therapy of insulin to the glimepiride and metformin combination and the addition of the glimepiride and metformin combination to insulin therapy will support the successful accomplishment of glycemic control through lowered HbA1c levels. Good to excellent efficacy and tolerability was observed in patients with T2DM across the age groups, early as well as long-standing disease, and those associated with comorbidities like hypertension, dyslipidemia, and coronary artery disease. Hence, this therapy is favorable for the effective management of T2DM.
